# Targeting Key Mechanisms of Nutraceuticals in Chronic Pain: A Narrative Review

**DOI:** 10.3390/nu18213488

**Published:** 2026-10-23

**Authors:** Vittorio Schweiger, Barbara Ganz, Erica Secchettin, Marta Nizzero, Luca Polati, Alessandro Picelli, Giustino Varrassi, Luca Dalle Carbonare, Leonardo Gottin, Alvise Martini, Enrico Polati

**Affiliations:** 1 Department of Surgery, Dentistry, Paediatrics and Gynaecology, Section of Anesthesia, Intensive Care and Pain Therapy, Verona University Hospital, 37134 Verona, Italy; erica.secchettin@univr.it (E.S.); marta.nizzero@aovr.veneto.it (M.N.); pola.luca@hotmail.it (L.P.); leonardo.gottin@univr.it (L.G.); alvise.martini@univr.it (A.M.); enrico.polati@univr.it (E.P.); 2 Pain Therapy Centre, Palagi Hospital, 50122 Firenze, Italy; barbara.ganz@uslcentro.toscana.it; 3 Department of Neurosciences, Biomedicine and Movement Sciences, University of Verona, 37134 Verona, Italy; alessandro.picelli@univr.it; 4 VIBRA Research Group, Paolo Procacci Foundation, 00193 Rome, Italy; giuvarr@gmail.com; 5 Department of Engineering for the Innovation Medicine, University of Verona, 37134 Verona, Italy; luca.dallecarbonare@univr.it

**Keywords:** chronic pain, nutraceuticals, neuroinflammation, oxidative stress, central sensitization, peripheral sensitization, pain pathophysiology

## Abstract

Chronic pain (CP) is a major cause of disability worldwide and reflects interactions among multiple pathophysiological mechanisms. Despite the availability of pharmacological therapies, many patients continue to experience inadequate pain control, supporting interest in complementary approaches. Nutraceuticals have been investigated as potential adjuncts because several compounds modulate biological pathways implicated in pain persistence. This narrative review provides a mechanism-based overview of nutraceuticals in CP across five interconnected domains: neuroinflammation, oxidative stress and mitochondrial dysfunction, central sensitization, peripheral sensitization, and descending inhibitory modulation. Literature was identified in PubMed/MEDLINE and the Cochrane Library, with the final update completed on 8 October 2026; preclinical mechanisms and clinical efficacy were evaluated separately. Earlier short-term trials of alpha-lipoic acid suggested symptomatic improvement in diabetic peripheral neuropathy, whereas a 2024 Cochrane review found little or no benefit after six months. Evidence syntheses suggest possible analgesic effects of palmitoylethanolamide in mixed chronic pain populations and of curcuminoids in knee osteoarthritis. Clinical studies of omega-3 fatty acids also suggest a possible reduction in pain intensity, although substantial heterogeneity across conditions, formulations, and study designs limits interpretation. Across compounds, mechanistic plausibility generally exceeds evidence of clinical efficacy and direct target engagement in humans. Future studies should prioritize standardized formulations, clinically relevant outcomes, adequate follow-up, safety assessment, and mechanistic biomarkers within phenotype-informed pain management.

## 1. Introduction

Chronic pain (CP) is now widely recognized as a major global health priority and a leading contributor to years lived with disability worldwide [1,2]. According to large epidemiological surveys, between 12% and 30% of the European population and approximately 20% of adults in the United States experience pain persisting for more than three months [3,4]. The Global Burden of Disease Study consistently ranks CP conditions among the leading causes of long-term disability, with profound socioeconomic implications [1]. Beyond nociception itself, CP is associated with impaired functional capacity, sleep disturbances, mood disorders, cognitive dysfunction, and reduced work productivity [2,3,4,5]. The bidirectional relationship between chronic pain and affective disorders further amplifies disability and healthcare utilization. The economic burden, including both direct medical costs and indirect productivity losses, is substantial and comparable to that of major chronic diseases such as diabetes and cardiovascular disease [2]. Despite the availability of multiple pharmacological classes-including nonsteroidal anti-inflammatory drugs (NSAIDs), acetaminophen, opioids, antidepressants, and anticonvulsants—therapeutic outcomes remain unsatisfactory. Large population-based surveys indicate that 40–60% of patients report inadequate pain control despite receiving standard treatment regimens [4]. This gap reflects not only limitations in efficacy and tolerability but also the biological complexity of chronic pain syndromes. Modern pain classification frameworks, including the 2020 revision of the International Association for the Study of Pain (IASP), recognize that CP often comprises overlapping nociceptive, neuropathic, and nociplastic components [6]. Peripheral tissue damage may coexist with central sensitization, neuroimmune activation, maladaptive synaptic plasticity, and impaired descending inhibitory pathways [7,8]. These mechanisms interact dynamically, forming a self-sustaining network in which inflammatory mediators, oxidative stress, altered neurotransmission, and dysfunctional pain modulatory circuits perpetuate nociceptive amplification even after resolution of the initial insult. Furthermore, concerns regarding the long-term safety of pharmacological therapies—particularly the gastrointestinal, renal, and cardiovascular risks associated with NSAIDs, as well as opioid misuse and dependence—have intensified interest in complementary strategies that target multiple biological pathways simultaneously [9,10]. Within this evolving therapeutic landscape, nutraceuticals—bioactive compounds derived from dietary sources with potential health benefits—have emerged as potential adjunctive therapeutic options [11]. However, most of the existing literature adopts a compound-centered approach, focusing on individual nutraceuticals without integrating them into the broader pathophysiological framework of chronic pain. This fragmented perspective limits both mechanistic understanding and clinical applicability. Importantly, the present review does not seek to establish superiority among nutraceuticals but rather to critically examine how different compounds converge on shared biological mechanisms involved in chronic pain. A mechanism-based integrative framework may therefore provide a more coherent strategy for evaluating the translational potential of nutraceutical interventions.

## 2. Methods

### 2.1. Literature Identification and Update

The literature relevant to the initial development of this narrative review was identified between April and May 2026, with searches focusing on chronic pain conditions, nutraceutical interventions, and biological mechanisms involved in pain persistence and chronification. Given the mechanism-based scope of the review, the literature was considered not only according to individual compounds or pain diagnoses, but also to its relevance to the main pathophysiological processes underlying CP. Following peer review, the search approach was retrospectively formalized and subsequently updated to improve transparency and reproducibility. The strategies reported in Supplementary Table S1 document this reconstructed approach and should not be interpreted as a verbatim record of the initial April–May 2026 searches. Three complementary search approaches were adopted. First, a broad search combined terms related to CP conditions with terms related to nutraceuticals, dietary supplements, and bioactive compounds. Second, a mechanism-oriented search combined CP terms with the five pathophysiological domains considered in this review: neuroinflammation, oxidative stress and mitochondrial dysfunction, central sensitization, peripheral sensitization, and impaired descending pain modulation. Third, compound-specific searches were performed for the individual nutraceuticals included in the review, with particular attention to evidence derived from randomized controlled trials, systematic reviews, and meta-analyses when available. PubMed/MEDLINE was used as the primary bibliographic database. The Cochrane Library was additionally consulted to identify relevant systematic reviews, particularly when evaluating clinical efficacy and longer-term treatment effects. Reference lists of relevant systematic reviews and key primary studies were also examined to identify additional publications pertinent to the mechanistic or clinical questions addressed in the review. The final literature update was completed on 8 October 2026. Searches were limited to publications in English. Pain terms covered CP and the main conditions discussed in the review (neuropathic pain, diabetic peripheral neuropathy, fibromyalgia, osteoarthritis, chronic low back pain and migraine); nutraceutical terms covered the generic terms above together with the name and common synonyms of each compound listed in Table 1 and Table 2. The search strings are reported in Supplementary Table S1. No review protocol was registered. Because the aim of the present work was to provide a mechanism-based narrative synthesis rather than an exhaustive systematic review, the search was not intended to support formal estimates of treatment efficacy or certainty of evidence. Instead, it was used to identify and critically contextualize the experimental and clinical literature relevant to the proposed biological targets and their potential translation into CP management.

### 2.2. Scope and Selection of Evidence

Studies were considered according to their relevance to one or more of the predefined pathophysiological domains and to their contribution to the mechanistic or clinical understanding of nutraceutical interventions in chronic pain. Evidence from experimental models was included when it provided biologically relevant information on nociceptive processing, neuroimmune signaling, oxidative and mitochondrial homeostasis, neuronal sensitization, or endogenous pain modulation. Such studies were used to support mechanistic interpretation and were not considered evidence of clinical analgesic efficacy. Clinical studies were considered when they evaluated pain-related outcomes in chronic pain conditions relevant to the scope of the review. Randomized controlled trials, controlled clinical studies, systematic reviews, and meta-analyses were preferentially considered when discussing clinical efficacy, whereas observational studies were used primarily to provide complementary translational or safety information. Where several systematic reviews addressed the same compound and condition, the most recent and comprehensive ones were preferred, and discordant conclusions were reported rather than reconciled (for example, for alpha-lipoic acid and ginger). Studies addressing acute pain were generally excluded from the clinical synthesis, although experimental studies using acute nociceptive paradigms could be retained when they provided direct information on a mechanism considered relevant to CP. Nutraceuticals were selected when two conditions were met: a biologically plausible relationship with at least one of the predefined pain mechanisms and the availability of relevant experimental and/or clinical evidence. The selection was therefore intended to identify representative compounds across the mechanistic domains rather than to provide an exhaustive catalogue of all dietary supplements or naturally derived compounds proposed for pain management. For the purposes of this review, the term “nutraceutical” was used operationally to encompass nutrients, naturally derived bioactive compounds, and dietary constituents administered as supplements or formulated preparations and investigated for biological actions potentially relevant to chronic pain. Where appropriate, supplementation aimed at correcting a documented nutritional deficiency was distinguished from the use of the same nutrient as a pain-modulating intervention in individuals without established deficiency. Records were screened, and the evidence was appraised by two authors (V.S. and E.P.). Disagreements were resolved by consensus, with a third author (E.S.) consulted when required.

### 2.3. Interpretation of Mechanistic and Clinical Evidence

Given the translational focus of the review, mechanistic plausibility, clinical efficacy, and evidence of target engagement in humans were considered as distinct dimensions. Mechanistic evidence was derived primarily from cellular and animal studies and was used to assess whether a compound could plausibly influence one or more biological processes involved in CP. Clinical efficacy was evaluated separately based on human studies reporting pain-related or functional outcomes. Evidence of target engagement was considered only when human data directly supported modulation of the proposed biological pathway or biomarker. The clinical literature was interpreted considering study design, sample size, treatment duration, consistency of findings, formulation and route of administration, comparator treatment, and clinically relevant methodological limitations. Particular weight was given to randomized controlled trials and systematic reviews or meta-analyses when available. For these syntheses, we relied on the risk-of-bias and certainty-of-evidence assessments reported by their authors, such as the Cochrane GRADE ratings for alpha-lipoic acid and acetyl-L-carnitine, and did not re-grade the primary studies. Importantly, mechanistic plausibility alone was not considered sufficient to infer clinical effectiveness. No formal GRADE assessment or validated evidence-certainty framework was applied, as this was beyond the scope of the narrative review. For the main compounds, study design, population, comparator, dose, formulation, treatment duration and clinical outcomes are summarized where reported in Supplementary Table S2. Studies authored by members of the present group were not used as the sole support for any statement on clinical efficacy and are identified as such in the text. Accordingly, the tables summarize indication-specific clinical findings and their principal limitations in descriptive terms, without applying categorical grades or implying recommendations for clinical practice.

## 3. Conceptual Framework

CP is increasingly conceptualized not as a single pathological entity but as an emergent property of interacting and self-reinforcing biological systems [7,8]. Neuroimmune activation, redox imbalance, mitochondrial dysfunction, altered excitatory–inhibitory neurotransmission, and impaired descending pain modulation constitute interdependent components of a dynamic pathological network. At both the spinal and supraspinal levels, activation of microglia and astrocytes leads to the release of pro-inflammatory cytokines and NF-κB-dependent transcriptional changes that enhance excitatory neurotransmission while reducing inhibitory tone [7,8,9,10,11,12]. Simultaneously, oxidative stress and mitochondrial dysfunction disrupt neuronal homeostasis and promote nociceptor sensitization [13]. At the peripheral level, inflammatory mediator release and activation of transient receptor potential (TRP) channels contribute to peripheral sensitization by increasing afferent nociceptive input [14]. Dysfunction of descending serotonergic and noradrenergic pathways further impairs endogenous pain inhibition, particularly in nociplastic disorders such as fibromyalgia syndrome (FMS) [15]. Importantly, these mechanisms do not operate in isolation. Neuroinflammation amplifies oxidative stress, oxidative stress enhances cytokine signaling, mitochondrial dysfunction disrupts synaptic transmission, and impaired descending inhibition facilitates central sensitization. Consequently, the persistence of CP reflects network-level dysregulation rather than a single molecular defect. Accordingly, this review organizes the available evidence on nutraceuticals into five interrelated mechanistic domains: neuroinflammation; oxidative stress and mitochondrial dysfunction; central sensitization; peripheral sensitization and nociceptor hyperexcitability; and impaired descending inhibitory modulation. Within each domain, we discuss the underlying molecular pathways together with nutraceutical agents supported by mechanistic rationale and available clinical evidence. While this mechanism-based framework facilitates biological interpretation, it should not be taken to imply equivalent clinical evidence across all nutraceuticals. The strength of translational support varies substantially among compounds, with robust randomized clinical evidence currently available for only a limited number of interventions. The discussed compounds, their proposed mechanisms, and the clinical contexts in which they have been investigated are summarized in Table 1 and Table 2. Study-level characteristics relevant to clinical interpretation are outlined in Supplementary Table S2. Clinical evidence was appraised descriptively by pain indication, independently of mechanistic plausibility. Descriptors used in the tables are qualitative author judgments and do not represent GRADE ratings or formal certainty-of-evidence estimates.

## 4. Key Pathophysiological Mechanisms as Nutraceutical Targets

### 4.1. Neuroinflammation

Neuroinflammation is increasingly recognized as a central driver of CP maintenance rather than a mere epiphenomenon of tissue injury. At the spinal and supraspinal levels, activation of microglia and astrocytes leads to the release of pro-inflammatory cytokines, including IL-1β, TNF-α, and IL-6, together with chemokines and reactive oxygen species (ROS). These mediators enhance excitatory glutamatergic neurotransmission, reduce inhibitory tone, and promote long-term potentiation-like changes within nociceptive pathways [7,11,14]. Importantly, glial–neuronal crosstalk contributes to both neuropathic and nociplastic pain states, including fibromyalgia syndrome (FMS) and chronic low back pain (CLBP). Neuroinflammation also interacts bidirectionally with oxidative stress and mitochondrial dysfunction, creating a self-amplifying loop that sustains central sensitization. This integrative view provides a rationale for the use of agents capable of modulating neuroinflammatory signaling without inducing systemic immunosuppression. Several nutraceuticals have demonstrated anti-neuroinflammatory properties. Curcumin has been shown to inhibit NF-κB activation and downstream cytokine transcription in microglial and macrophage cultures [16]. In a rat model of chronic constriction injury, curcumin attenuated mechanical allodynia and reduced spinal BDNF and COX-2 expression [17]; modulation of glial signaling has been proposed as a contributing mechanism [18]. However, most human studies rely on symptomatic endpoints, and direct biomarker confirmation of NF-κB modulation in vivo remains limited. Another important limitation to the clinical application of curcumin in CP is its poor absorption, rapid metabolism, and limited systemic bioavailability, which result in low plasma and tissue concentrations. Several studies have reported improved pharmacokinetic profiles with enhanced-delivery formulations, although increased exposure does not by itself establish analgesic efficacy [19]. In knee osteoarthritis, systematic reviews of randomized trials have reported improvements in pain and function with turmeric extracts or curcuminoids over short treatment periods; however, heterogeneity, variable risk of bias, and formulation differences limit the certainty and generalizability of these findings [20,21]. Among nutraceuticals, palmitoylethanolamide (PEA) has a substantial mechanistic rationale within the neuroinflammatory domain. PEA primarily acts through direct activation of the nuclear receptor peroxisome proliferator-activated receptor-α (PPAR-α), thereby modulating mast cell degranulation and microglial reactivity and restoring neuroimmune homeostasis rather than simply suppressing neuroinflammation [22,23]. However, native PEA exhibits poor oral absorption because of its large particle size, which markedly limits its tissue distribution and bioavailability. To overcome these limitations, micronized (mPEA; particle size 2–10 μm) and ultra-micronized (umPEA; particle size 0.8–6 μm) formulations have been developed to enhance bioavailability and improve biological activity at the cellular level [24,25]. A recent narrative review by Nobili et al. further emphasized that the clinical interpretation of PEA should account for formulation, summarizing pharmacokinetic, preclinical, and clinical data on umPEA and its use mainly as an adjunct to conventional analgesic treatment [26]. Furthermore, mPEA and umPEA combined with natural compounds such as the antioxidant polydatin have demonstrated synergistic effects and enhanced biological efficacy [27]. Clinical data on PEA are heterogeneous and cannot be generalized across indications; neuropathic pain and FMS should not be treated as interchangeable. Systematic reviews and meta-analyses by groups not involved in the present work reported lower pain scores with PEA than with control in mixed chronic pain populations [28,29], but formulations, doses, indications and comparators varied considerably, and evidence specific to FMS is limited. Earlier syntheses and observational reports, some authored by members of the present group, point in the same direction but are more exposed to bias [30,31,32]. A 2024 systematic review and meta-analysis from members of the present author group evaluated nine studies involving 742 patients treated with micron-size PEA for at least 60 days and reported further pain reduction beyond the first month. Because eight of the nine included studies were uncontrolled and clinical heterogeneity was high, these findings are more informative regarding treatment duration than comparative efficacy [33]. Direct human evidence of neuroimmune target engagement remains scarce. Among polyunsaturated fatty acids (PUFAs), eicosapentaenoic acid (EPA) and docosahexaenoic acid (DHA), collectively known as omega-3 fatty acids, exert anti-inflammatory effects largely through the biosynthesis of specialized pro-resolving mediators (SPMs), including resolvins and protectins [34]. At the central level, experimental evidence indicates that omega-3 polyunsaturated fatty acid supplementation can attenuate neuroinflammation and modulate microglial polarization through the SIRT1-mediated deacetylation of the HMGB1/NF-κB pathway following traumatic brain injury [35]. In animal models, EPA also reduced FMS-like pain by modulating microglial and astrocytic activation as well as Toll-like receptor 4 signaling in the cerebellum [36]. Furthermore, resolvin D1 reduced spinal IL-1β expression and mechanical hypersensitivity in rat models [36]. Regarding omega-6 fatty acids, the arachidonic acid (AA) and linoleic acid (LA) metabolic pathways appear to play important roles in modulating ion channel activity in peripheral sensory neurons. In particular, accumulating evidence from animal studies suggests that metabolites of AA (epoxyeicosatrienoic acids, EETs, and dihydroxyeicosatrienoic acids, DHETs) and LA (epoxyoctadecenoic acids, EpOMEs, and dihydroxyoctadecenoic acids, DiHOMEs) contribute to the regulation of persistent pain and NP while also participating in pathways that limit neuroinflammatory processes [37]. Resveratrol modulates inflammatory signaling through activation of SIRT1 and inhibition of NF-κB [38]. In experimental models of neuropathic pain, SIRT1 upregulation is associated with reduced cytokine expression and attenuated behavioral hypersensitivity. Nevertheless, despite promising preclinical findings, clinical evidence supporting the efficacy of resveratrol in CP remains limited, and further well-designed randomized controlled trials are needed [39]. Poor bioavailability and heterogeneity among resveratrol formulations further limit extrapolation from experimental data, and the human supplementation data available come mainly from non-pain populations, such as a high-dose trial in obese men [40]. Acetyl-L-carnitine (ALC), the acetylated derivative of L-carnitine, has also demonstrated anti-neuroinflammatory properties that may be beneficial in CP. Studies cited here report neuroprotective or anti-inflammatory effects of ALC in Parkinsonian rats and experimental spinal cord injury, rather than in chronic-pain models [41,42]. Evidence from experimental NP models has also been reported [43]. Alpha-lipoic acid (ALA), also known as thioctic acid, is an essential cofactor for several mitochondrial enzymatic complexes involved in cellular energy metabolism. Although it is primarily recognized for its antioxidant properties, ALA has also demonstrated anti-neuroinflammatory effects in experimental models of complex regional pain syndrome (CRPS) [44]. Overall, neuroinflammation represents one of the most biologically plausible targets for nutraceutical intervention. Nevertheless, despite consistent mechanistic rationale, clinical evidence remains heterogeneous, including for PEA, and cannot be inferred from anti-neuroinflammatory activity alone. Future studies integrating mechanistic biomarkers with clinical outcomes will be essential to confirm target engagement in humans for most nutraceuticals. Clinical evidence should be considered separately: a meta-analysis of 41 randomized trials (3759 participants) reported an overall reduction in CP intensity (SMD −0.55; 95% CI −0.76 to −0.34), with substantial heterogeneity (*I*^2^ = 87%) and no statistically clear benefit in the OA subgroup [45]. A separate rheumatoid arthritis meta-analysis found a small, uncertain effect on pain, supporting condition-specific interpretation [46].

### 4.2. Oxidative Stress and Mitochondrial Dysfunction

Oxidative stress is deeply intertwined with the pathophysiology of CP. Excessive production of reactive oxygen and nitrogen species (ROS/RNS) contributes to lipid peroxidation, mitochondrial membrane depolarization, and neuronal apoptosis [13]. In diabetic peripheral neuropathy (DPN), oxidative damage impairs axonal transport and nerve conduction, whereas in fibromyalgia syndrome (FMS), mitochondrial dysfunction and altered energy metabolism have been proposed as contributing mechanisms [13,47]. Importantly, oxidative stress amplifies neuroinflammation, while inflammatory cytokines further enhance ROS production, thereby reinforcing a self-perpetuating pathogenic loop [48]. Several nutraceuticals have been investigated for their antioxidant properties and potential therapeutic role in CP. Alpha-lipoic acid (ALA) acts both as a direct scavenger of ROS and as a regenerator of endogenous antioxidants, including glutathione [49]. The SYDNEY 2 trial reported short-term symptomatic improvement with oral ALA in DPN [50]. In contrast, a 2024 Cochrane review of trials with at least six months of treatment concluded that ALA probably has little or no effect on neuropathic symptoms at six months; this finding came from a single trial of 330 participants (moderate-certainty evidence), within a review of three placebo-controlled trials with treatment lasting 6 to 48 months [51]. The different follow-up periods and study populations preclude extrapolating early symptomatic changes to sustain analgesic benefit. Coenzyme Q10 (CoQ10) is an essential component of the mitochondrial electron transport chain and plays a pivotal role in cellular energy production [52]. In its reduced form, ubiquinol, CoQ10 also acts as a potent lipid-soluble antioxidant, recycling other antioxidants such as vitamins E and C while limiting both the initiation and propagation of ROS-mediated oxidative damage [53]. Following encouraging preclinical findings demonstrating prevention of DPN development in diabetic animal models, one placebo-controlled trial in painful DPN (112 participants) reported improved pain intensity with CoQ10 300 mg/day added to pregabalin 150 mg/day over eight weeks; the result does not establish efficacy as monotherapy [54,55]. In FMS, a small clinical study reported improvements in oxidative stress biomarkers and pain severity [56], which is consistent with, but does not prove, the hypothesis that enhancement of mitochondrial function may modulate nociplastic pain mechanisms. Resveratrol has also been shown to activate Nrf2-dependent antioxidant transcription pathways, thereby enhancing endogenous antioxidant defenses [57]. However, clinical evidence supporting its analgesic efficacy remains limited [58]. Selenium and vitamin C contribute to endogenous antioxidant defense systems, although most evidence supporting their use in pain conditions relates to correction of nutritional deficiencies rather than to direct analgesic effects [59,60]. Curcumin also exerts antioxidant effects in experimental models by increasing the activity of antioxidant enzymes, including superoxide dismutase (SOD) and glutathione peroxidase (GPx), while reducing lipid peroxidation [61]. In humans, changes in serum cytokine concentrations have been reported with curcuminoids in metabolic syndrome [62], which is not a pain population. Whether these antioxidant effects translate into analgesia in DPN remains uncertain [61]. In a 16-week placebo-controlled trial, nanocurcumin did not improve pain or neuropathic outcomes in patients with DPN [63]. Antioxidant properties have also been reported for tryptophan (TR), acetyl-L-carnitine (ALC), β-caryophyllene (BCP), trace elements such as zinc (Zn^2+^) and selenium (Se), and vitamin C (VitC), although clinical evidence remains limited [59,60,64,65,66,67]. Taken together, antioxidant nutraceuticals may indirectly reduce pain by restoring cellular homeostasis rather than acting as direct analgesics.

### 4.3. Central Sensitization

Central sensitization is characterized by enhanced responsiveness of nociceptive neurons within the dorsal horn of the spinal cord and supraspinal structures [8]. Its underlying mechanisms include *N*-methyl-D-aspartate (NMDA) receptor overactivation, increased glutamatergic neurotransmission, reduced GABAergic inhibition, and long-term synaptic plasticity [68]. Several nutraceuticals have been proposed to target these mechanisms. Magnesium (Mg^2+^) physiologically blocks NMDA receptor channels in a voltage-dependent manner [69]. Reduced magnesium levels may facilitate NMDA receptor overactivation and neuronal hyperexcitability. A systematic review reported that magnesium supplementation showed the greatest benefit in migraine prophylaxis and, to a lesser extent, in certain NP conditions, although dosing regimens and routes of administration vary considerably across studies [70]. In preclinical models, ALC increases metabotropic glutamate receptor 2 (mGlu2) expression through epigenetic mechanisms, and activation of group II mGlu receptors reduces NP behavior [71]. A meta-analysis of randomized controlled trials (RCTs) reported a moderate reduction in pain with ALC in peripheral NP, particularly in DPN [72]; however, the Cochrane review restricted to DPN rated the certainty of the evidence for pain outcomes as very low [73]. Overall, these compounds may contribute to restoring the balance between excitatory and inhibitory neurotransmission involved in central sensitization. Compared with neuroinflammation and oxidative stress, nutraceutical interventions targeting central sensitization remain comparatively underdeveloped.

### 4.4. Peripheral Sensitization

Peripheral sensitization represents a key mechanism underlying CP. Following tissue injury, the release of pro-inflammatory mediators—including prostaglandins, bradykinin, cytokines, chemokines like chemokine C-C motif ligand 2 (CCL2), and nerve growth factor (NGF) lowers the activation threshold of peripheral nociceptors, resulting in hyperalgesia and allodynia [74]. Dysregulation of ion channels, including transient receptor potential vanilloid 1 (TRPV1), voltage-gated sodium channels (NaV1.7 and NaV1.8), and acid-sensing ion channels (ASICs), is considered one of the pivotal mechanisms underlying peripheral sensitization [13]. Peripheral sensitization also contributes to NP pathophysiology through the upregulation of sodium channels and increased expression of neuropeptides such as substance P (SP) and calcitonin gene-related peptide (CGRP) [75]. Several nutraceuticals have been proposed to modulate these mechanisms in both inflammatory and NP scenarios. Boswellic acids (BAs), a group of pentacyclic triterpenes derived from *Boswellia serrata*, exert anti-inflammatory effects through inhibition of the complement system, 5-lipoxygenase (5-LOX), and microsomal prostaglandin E_2_ synthase-1 (mPGES-1) [76,77]. In osteoarthritis (OA), both preclinical and clinical studies suggest that BAs may alleviate pain and improve joint function, although evidence of structural disease modification or direct target engagement in patients is lacking [78]. A systematic review of nine randomized trials (712 participants) found improvements in knee OA symptoms, but results may vary across standardized extracts [79]. Ginger, an herbaceous plant belonging to the *Zingiberaceae* family, contains numerous bioactive nonvolatile compounds with anti-inflammatory properties that have been associated with pain reduction [80]. The relationship between TRPV1 activation and analgesia is complex. Initial channel activation may be followed by receptor desensitization depending on concentration and duration of exposure, whereas sustained activation in injured or inflamed tissue may maintain peripheral sensitization [14]; therefore, the clinical analgesic effects of ginger should not be directly attributed to TRPV1 agonism alone. The antinociceptive effect of ginger extract in rats was shown in an acute thermal model [81], which does not reproduce CP, and this mechanistic hypothesis remains distinct from clinical efficacy outcomes. Meta-analyses of randomized trials have reported small improvements in OA symptoms, although conclusions differ between syntheses and methodological limitations persist [82,83,84]. Other nutraceuticals that may influence peripheral sensitization include β-caryophyllene (BCP), PEA, and zinc (Zn^2+^), although their clinical relevance in CP remains to be established [22,85,86]. Overall, targeting peripheral sensitization appears to provide greater clinical benefit in inflammatory musculoskeletal pain than in NP conditions. This observation further emphasizes the importance of matching nutraceutical interventions to the predominant pain phenotype rather than considering CP as a homogeneous clinical entity.

### 4.5. Descending Inhibitory Modulation

Descending serotonergic and noradrenergic pathways originating in the brainstem exert inhibitory control over spinal nociceptive transmission [15]. Dysfunction of these descending inhibitory pathways has been implicated in several CP conditions, particularly nociplastic disorders like FMS. Nevertheless, the contribution of impaired descending pain modulation in the pathophysiology of FMS remains controversial [87,88,89]. Tryptophan (TR) is the metabolic precursor of several bioactive compounds, including serotonin (5-hydroxytryptamine, 5-HT). Within the spinal cord, serotonergic pathways exert complex modulatory effects on nociceptive transmission, producing either inhibition or facilitation depending on the receptor subtype involved [90]. Although tryptophan and 5-hydroxytryptophan have been investigated in several painful conditions, including migraine, FMS, small fiber neuropathy (SFN), and musculoskeletal pain, available clinical evidence is insufficient to establish their efficacy for routine CP management. Omega-3 fatty acids modulate inflammatory signaling in experimental systems [34,36]. Clinical findings for omega-3 fatty acids remain heterogeneous across pain conditions (clinical synthesis requires an additional bibliographic entry), and direct human evidence that supplementation restores descending inhibitory control is lacking. Vitamin D receptors are distributed throughout the human brain [91]. A Cochrane review of 10 randomized trials involving 811 participants found no consistent analgesic benefit from vitamin D supplementation on CP states [92]. A more recent systematic review and meta-analysis focusing on FMS suggested possible improvements in pain intensity and disease-related impact, but the pooled estimates were based on small datasets with substantially high heterogeneity across studies [93]. Differences in baseline vitamin D status and supplementation regimens limit generalizability; correction of documented deficiency should be distinguished from supplementation for analgesia in vitamin D-sufficient patients. Overall, nutraceutical modulation of descending inhibitory pathways remains one of the least clinically validated approaches, and currently available evidence does not support routine supplementation outside selected clinical contexts, particularly nutritional deficiency states.

## 5. Critical Issues and Future Perspectives

This mechanism-based narrative review indicates that the biological plausibility of nutraceutical modulation of nociceptive pathways frequently exceeds the strength of the corresponding clinical evidence. While many nutraceuticals consistently modulate key pathways involved in CP in experimental models, demonstration of comparable target engagement in humans remains limited. This translational gap should be considered when interpreting current evidence and designing future clinical studies. In particular, variability in bioavailability and gastrointestinal (GI) tract absorption can substantially influence their clinical efficacy in humans, and classification systems based on solubility, absorption, and transport have been proposed to better predict their pharmacokinetic behavior [94]. This approach is particularly relevant because the bioavailability of many nutraceuticals is inherently limited but may be improved through technological innovations, including nanotechnology-based delivery systems, designed to enhance bioaccessibility and biotransformation [95,96]. Drug–nutraceutical interactions may also influence both efficacy and safety, particularly in patients receiving multiple medications. Accordingly, the need for evidence-based guidelines to optimize the use of nutraceuticals in polypharmacy settings has been increasingly emphasized, underscoring the importance of interdisciplinary collaboration in identifying and managing potential interactions [97]. Additional challenges include the standardization of plant extracts, variability in the concentration of active constituents, manufacturing quality, and regulatory issues, all of which limit the reproducibility and generalizability of clinical findings and complicate the assessment of both efficacy and safety [98]. Finally, potential benefits of nutraceutical combinations require validation in well-defined pain phenotypes [96,99,100].

### 5.1. Limitations of Clinical Evidence

Beyond pharmacokinetic limitations, clinical literature is affected by small samples, short treatment periods, inconsistent diagnostic criteria and pain outcome measures, and variable concomitant analgesic regimens. Placebo responses and selective publication may exaggerate apparent effects, particularly in small trials. In addition, trials commonly report changes in pain intensity without prespecified clinically relevant differences or functional endpoints. Direct evidence of target engagement in patients is uncommon: a reduction in pain does not establish that the compound modulated microglial signaling, NF-κB activity, oxidative stress, or another proposed pathway at the exposure achieved clinically. These limitations preclude causal attribution of analgesic effects to the mechanisms demonstrated in experimental models. The narrative design of this review has its own limitations: no protocol was registered, only PubMed/MEDLINE and the Cochrane Library were searched, and study selection did not follow a prespecified systematic procedure, so relevant studies may have been missed. The conclusions should be read as an interpretive synthesis rather than a quantitative estimate of effect.

### 5.2. Safety and Clinical Applicability

The use of nutraceuticals as adjuncts to multimodal pain treatment requires compound- and formulation-specific assessment of tolerability, contraindications, interactions, and dose. Gastrointestinal adverse effects may occur with several agents, including magnesium salts, ALA, and botanical extracts. Curcumin and ginger preparations warrant review of potential bleeding-related interactions in patients receiving antithrombotic drugs, while highly bioavailable curcumin products have also been associated with rare reports of liver injury [101]. Omega-3 preparations can cause gastrointestinal symptoms and should be reviewed in patients receiving antithrombotic therapy or high-dose products. Magnesium accumulation is a particular concern in advanced renal impairment; excessive vitamin D, selenium, or zinc intake can cause toxicity, and tolerable upper intake levels have been defined by regulatory agencies, for example, for vitamin D [102]. These considerations should not be interpreted as equivalent risks across all formulations or doses. For PEA, short trials generally report good tolerability, but longer-term and interaction data remain limited. Correction of a documented nutritional deficiency must be distinguished from supplementation intended to exert analgesic effects in nutritionally sufficient individuals. In routine pain practice, nutraceuticals use should be documented alongside prescription and non-prescription medications, especially in patients with polypharmacy or relevant comorbidity. Specific safety considerations and gaps in the underlying evidence are summarized in Supplementary Table S2. Findings obtained with a specific dose, route, standardized extract, or delivery system should not be extrapolated to other formulations of the same compound without supporting pharmacokinetic or clinical evidence. Supplementary Table S2 summarizes the trial-level information that could be verified and explicitly identifies unreported details.

### 5.3. Practical Considerations for Pain Practice

The evidence reviewed does not support replacing guideline-recommended treatment with nutraceuticals. When one of these agents is considered as an adjunct, the data suggest a few practical points. The pain phenotype should match the setting in which the compound has actually been studied, for example knee OA for curcuminoids and turmeric extracts [20,103], or DPN for adjunctive CoQ10 [55]; extrapolation to other phenotypes is not supported. Preparation, dose, and duration with published data should be chosen rather than a generic product. The expected benefit and the time frame should be agreed with the patient beforehand, using a prespecified outcome such as a reduction in pain intensity of at least 30% or a clinically relevant functional improvement [104], and the product should be stopped if this is not reached. Because most trials last only a few weeks, an early benefit should not be assumed to persist, as the experience with ALA shows [50,51]. Vitamin D status and other deficiencies should be checked and corrected when relevant, since this is a different intervention from supplementation in sufficient patients, and all supplements should be recorded together with the other medications.

## 6. Conclusions

While several nutraceuticals influence neuroimmune, redox and neuronal pathways in experimental systems, demonstration of clinical efficacy and direct human target engagement remains limited and condition-dependent. In diabetic peripheral neuropathy, earlier short-term findings for alpha-lipoic acid must be balanced against the 2024 Cochrane evidence of little or no symptomatic benefit after six months. For palmitoylethanolamide, curcumin, and other agents, clinical signals require interpretation considering study quality, heterogeneous pain phenotypes, and formulation-specific exposure. Nutraceuticals should therefore be regarded as potential adjuncts rather than substitutes for evidence-based multimodal pain management. Future trials should prioritize adequate follow-up, standardized preparations, patient-relevant outcomes, safety reporting, and mechanistic biomarkers.

**Table 1:** Nutraceuticals with clinical studies relevant to chronic pain: qualitative, indication-specific appraisal.

Nutraceutical	Pharmacological Class	Mechanisms Involved in Nociceptive Modulation	Proposed Primary Mechanism	Investigated Pain Conditions	Qualitative Clinical Evidence
Alpha-lipoic acid (ALA)	Enzymatic cofactor	ROS scavenging; regeneration of endogenous antioxidants (e.g., glutathione); reduction in oxidative stress and neuroinflammation; improvement of mitochondrial function	Antioxidant activity	DPN [50,51]	Short-term DPN symptom improvement reported [50]; Cochrane 2024 found little or no benefit at ≥6 months [51].
Palmitoylethanolamide (PEA)	Bioactive lipid (ALIamide)	Activation of PPAR-α; modulation of mast cell degranulation and microglial activation; restoration of neuroimmune homeostasis; reduction in peripheral and central sensitization	Neuroinflammation reduction	Mixed chronic pain; NP; FMS [28,29,30,31,32,33]	Analgesic signal in mixed chronic pain RCTs, with heterogeneous formulations, indications, and comparators; FMS-specific certainty remains limited [28,29]. An author-group meta-analysis of predominantly uncontrolled studies suggests additional pain reduction with micron-size PEA beyond the first month but does not establish comparative efficacy [33].
Curcumin	Polyphenol	NF-κB inhibition; reduction in pro-inflammatory cytokines (IL-1β, TNF-α, IL-6); antioxidant activity; modulation of microglial activation and nociceptive sensitization	Antioxidant activity	OA [20,21,103]; DPN (negative placebo-controlled trial [63]); NP (predominantly preclinical) [61,62]	Short-term pain/function benefit reported in knee OA reviews; formulation and study heterogeneity limit inference [20,21,103].
Omega-3 (EPA/DHA)	Polyunsaturated fatty acids (PUFAs)	Biosynthesis of resolvins and protectins; suppression of microglial activation; modulation of SIRT1–HMGB1–NF-κB signaling; reduction in neuroinflammation	Inflammation reduction	Mixed chronic pain populations in RCTs [45]; rheumatoid arthritis [46]; FMS-like pain in experimental models [36]	Pooled RCT signal varies by pain phenotype, dose and duration; substantial heterogeneity (*I*^2^ = 87%) [45]; RA-specific effect uncertain [46].
Coenzyme Q10 (CoQ10)	Antioxidant; mitochondrial cofactor	Support of mitochondrial electron transport chain; reduction in oxidative stress; stabilization of mitochondrial membranes; modulation of nociceptive sensitization	Antioxidant activity	FMS; DPN [54,55,56]	One adjunctive DPN RCT with pregabalin; limited replication and follow-up [55].
Magnesium (Mg^2+^)	Essential mineral	Voltage-dependent NMDA receptor block; reduction in neuronal hyperexcitability; modulation of central sensitization	NMDA receptor block	Migraine; NP; nociplastic pain [70]	Clinical data mainly concern migraine prevention; limited evidence for other chronic pain phenotypes [70].
Ginger (gingerols)	Phenolic compound	Modulation of TRPV1 channels; reduction in inflammatory mediators; regulation of peripheral nociceptor activity	Inflammation reduction	OA; musculoskeletal pain [80,81]	Small and inconsistent OA symptom effects across meta-analyses; no clinical TRPV1 target confirmation [80,81].
Acetyl-L-carnitine (ALC)	Amino acid derivative	Epigenetic upregulation of mGlu2 receptors; reduction in glutamate release; anti-neuroinflammatory and neuroprotective effects	Epigenetic upregulation	DPN; NP [41,42,43,73]	Some neuropathic pain trials report benefit, but Cochrane assessment of DPN pain evidence is very uncertain [73].
Vitamin D	Fat-soluble vitamin; secosteroid hormone	Vitamin D receptor-mediated regulation of inflammatory cytokines and monoaminergic pathways; modulation of neuroimmune responses	Inflammation reduction	FMS, in vitamin D-deficient patients [92,93]	Possible benefit in selected fibromyalgia populations, but small studies and substantial heterogeneity limit certainty; no consistent benefit across chronic pain conditions [92,93].
Boswellic acids (BAs)	Pentacyclic triterpene	Inhibition of 5-lipoxygenase (5-LOX), complement activation, and microsomal prostaglandin E2 synthase-1 (mPGES-1); reduction in peripheral inflammation	Inflammation reduction	OA; inflammatory musculoskeletal pain [78]	Knee OA trials suggest symptom improvement; evidence depends on standardized extract and trial quality [78].

Note. Clinical descriptions are indication-specific, not formal grades of certainty; mechanistic findings do not establish analgesic efficacy. Abbreviations: ALA, alpha-lipoic acid; ALC, acetyl-L-carnitine; BAs, Boswellic acids; CB2, cannabinoid receptor 2; CGRP, calcitonin gene-related peptide; CoQ10, coenzyme Q10; CP, chronic pain; CRPS, complex regional pain syndrome; DHA, docosahexaenoic acid; DPN, diabetic peripheral neuropathy; EPA, eicosapentaenoic acid; FMS, fibromyalgia syndrome; mGlu2, metabotropic glutamate receptor 2; NF-κB, nuclear factor kappa B; NMDA, *N*-methyl-D-aspartate; NP, neuropathic pain; OA, osteoarthritis; PEA, palmitoylethanolamide; PPAR-α, peroxisome proliferator-activated receptor alpha; PUFAs, polyunsaturated fatty acids; ROS, reactive oxygen species; SIRT1, sirtuin 1; TRPV1, transient receptor potential vanilloid 1; 5-LOX, 5-lipoxygenase; mPGES-1, microsomal prostaglandin E2 synthase-1.

**Table 2:** Nutraceuticals supported mainly by preliminary clinical or experimental evidence.

Nutraceutical	Pharmacological Class	Mechanisms Involved in Nociceptive Modulation	Proposed Primary Mechanism	Investigated Pain Conditions	Qualitative Clinical Evidence
Resveratrol	Polyphenolic stilbene	Activation of SIRT1 and Nrf2 pathways; inhibition of NF-κB signaling; reduction in inflammatory cytokine production and oxidative stress	Inflammation reduction	Neuropathic pain (experimental models) [38]; no established clinical analgesic indication [39]	Predominantly experimental evidence; clinical analgesic efficacy unconfirmed.
Vitamin C	Water-soluble vitamin	ROS reduction; support of antioxidant systems; modulation of inflammatory responses	Antioxidant activity	CRPS; oxidative stress-related pain conditions [60]	Limited clinical evidence for selected indications; CRPS prevention is distinct from treatment of established CP.
Selenium (Se)	Trace mineral	Component of antioxidant selenoproteins (e.g., glutathione peroxidases); reduction in oxidative stress	Antioxidant activity	CP associated with deficiency states [59]	Evidence concerns nutritional biology/deficiency; analgesic benefit not established.
β-caryophyllene (BCP)	Sesquiterpene	Activation of cannabinoid CB2 receptors; modulation of neuroinflammation and peripheral sensitization	CB2 agonism	Inflammatory CP and NP [66,85]	Mechanistic and animal evidence; convincing clinical pain trials lacking.
Zinc (Zn^2+^)	Trace mineral	Regulation of immune responses and ion channel function; possible modulation of neuroinflammation	Neuroinflammation reduction	CP associated with deficiency states [67]	Preclinical evidence and deficiency-related rationale; clinical analgesic efficacy uncertain.
Tryptophan (TR)	Essential amino acid	Precursor of serotonin (5-HT); modulation of descending serotonergic pain pathways; influence on inhibitory/facilitatory nociceptive control	Descending pathways activation	FMS; migraine; SFN; musculoskeletal pain [64,90]	Limited clinical evidence; insufficient basis for routine use in chronic pain.

Note. Clinical descriptions are indication-specific, not formal grades of certainty. Abbreviations: BCP, beta-caryophyllene; CB2, cannabinoid receptor 2; CP, chronic pain; CRPS, complex regional pain syndrome; FMS, fibromyalgia syndrome; NF-κB, nuclear factor kappa B; NP, neuropathic pain; Nrf2, nuclear factor erythroid 2-related factor 2; ROS, reactive oxygen species; Se, selenium; SFN, small-fiber neuropathy; SIRT1, sirtuin 1; TR, tryptophan; 5-HT, 5-hydroxytryptamine; Zn, zinc.

## Data Availability

The data presented in this study are available from publicly accessible datasets. The sources of these data are cited in the manuscript.

## Supplementary Materials

The supporting information can be downloaded at https://www.mdpi.com/article/10.3390/nu18213488/s1. Table S1: Reconstructed PubMed search strategy; Table S2: Clinical Evidence and practical considerations.

## Abbreviations

The following abbreviations are used in this manuscript:

ALC	acetyl-L-carnitine
ALA	alpha-lipoic acid
AA	arachidonic acid
BAs	Boswellic acids
BCP	β-caryophyllene
CCL2	C-C motif chemokine ligand 2
CLBP	chronic low back pain
CP	chronic pain
CoQ10	coenzyme Q10
CGRP	calcitonin gene-related peptide
DHA	docosahexaenoic acid
DPN	diabetic peripheral neuropathy
EETs	epoxyeicosatrienoic acids
EPA	eicosapentaenoic acid
EpOMEs	epoxyoctadecenoic acids
FMS	fibromyalgia syndrome
GI	Gastrointestinal
GPx	glutathione peroxidase
LA	linoleic acid
mPEA	micronized palmitoylethanolamide
NaVs	voltage-gated sodium channels
NF-κB	nuclear factor kappa B
NMDA	*N*-methyl-D-aspartate
NP	neuropathic pain
OA	osteoarthritis
PEA	palmitoylethanolamide
PUFAs	polyunsaturated fatty acids
ROS	reactive oxygen species
RNS	reactive nitrogen species
SP	substance P
SPMs	specialized pro-resolving mediators
TR	tryptophan
TRP	transient receptor potential
TRPV1	transient receptor potential vanilloid 1
umPEA	ultra-micronized palmitoylethanolamide
