# Supplementary material for: Targeting Key Mechanisms of Nutraceuticals in Chronic Pain: A Narrative Review
Source: Nutrients. 2026 Oct 23;18(21):3488. doi: 10.3390/nu18213488 (PMC13765127; doi:10.3390/nu18213488)
Supplement: Supplementary file 1 [file nutrients-18-03488-s001.zip › nutrients-4602529-supplementary.pdf]

Supplementary Table S1. Reconstructed PubMed search strategy

The following three complementary PubMed/MEDLINE search strategies were retrospectively formalized during manuscript revision. They describe the approach used to structure the literature update and are not presented as exact records of the initial April–May 2026 searches. The original search history and retrieval counts were not available for retrospective verification.

S1-A. Pain conditions and nutritional interventions

("Chronic Pain"[MeSH Terms] OR "chronic pain"[Title/Abstract] OR "neuropathic pain"[Title/Abstract] OR fibromyalgia[Title/Abstract] OR osteoarthritis[Title/Abstract] OR "diabetic neuropathy"[Title/Abstract]) AND ("Dietary Supplements"[MeSH Terms] OR nutraceutical*[Title/Abstract] OR "dietary supplement"[Title/Abstract] OR "bioactive compound"[Title/Abstract] OR "natural compound"[Title/Abstract])

S1-B. Mechanistic domains

("chronic pain"[Title/Abstract] OR "neuropathic pain"[Title/Abstract] OR fibromyalgia[Title/Abstract] OR osteoarthritis[Title/Abstract]) AND (neuroinflammation[Title/Abstract] OR "oxidative stress"[Title/Abstract] OR "mitochondrial dysfunction"[Title/Abstract] OR "central sensitization"[Title/Abstract] OR "peripheral sensitization"[Title/Abstract] OR "descending pain modulation"[Title/Abstract]) AND (nutraceutical*[Title/Abstract] OR supplement*[Title/Abstract] OR "bioactive compound"[Title/Abstract])

S1-C. Compound-specific clinical retrieval

("chronic pain"[Title/Abstract] OR "neuropathic pain"[Title/Abstract] OR fibromyalgia[Title/Abstract] OR osteoarthritis[Title/Abstract] OR "diabetic peripheral neuropathy"[Title/Abstract]) AND (palmitoylethanolamide[Title/Abstract] OR "alpha-lipoic acid"[Title/Abstract] OR curcumin[Title/Abstract] OR "omega-3"[Title/Abstract] OR "coenzyme Q10"[Title/Abstract] OR "acetyl-L-carnitine"[Title/Abstract] OR magnesium[Title/Abstract] OR Boswellia[Title/Abstract] OR ginger[Title/Abstract] OR resveratrol[Title/Abstract] OR "vitamin D"[Title/Abstract] OR selenium[Title/Abstract] OR zinc[Title/Abstract] OR tryptophan[Title/Abstract] OR "beta-caryophyllene"[Title/Abstract])

Search scope: chronic pain conditions, five mechanistic domains and the individual nutraceuticals discussed in the narrative review. Search strings are presented for methodological transparency and reproducibility; no retrospective record counts or screening totals are inferred.

Supplementary Table S2. Clinical evidence and practical considerations

Trial-level characteristics are provided where supported by the cited publication. NR indicates information not reported in the source examined; NA, not applicable. Systematic reviews summarize heterogeneous trials and should not be interpreted as prescribing a single formulation or dosing regimen.

Agent / condition	Study type / design (N)	Evidence type; certainty as reported by the authors of the source	Formulation / route	Dose	Duration	Comparator / co-therapy	Primary outcome / findings	Limitations and safety	Adverse events reported in the source	Reference
ALA / DPN	SYDNEY 2; randomized placebo-controlled trial	Primary RCT (single trial); certainty not graded	Oral ALA	600, 1200 or 1800 mg/day	5 weeks	Placebo	Neuropathic symptom scores improved in short-term trial	Short duration; does not demonstrate sustained benefit	Dose-related nausea, vomiting and vertigo were reported; adverse events were more frequent with 1200 and 1800 mg/day than with 600 mg/day (SYDNEY 2).	[50]
ALA / DPN	2024 Cochrane; 3 placebo-controlled RCTs, 816 analysed participants	Systematic review (Cochrane); 6-month symptom outcome: moderate	Oral ± initial intravenous ALA	Regimens varied; ≥600 mg/day in included trials	6–48 months across the included trials; six-month symptom outcome	Placebo	Probably little or no effect on DPN symptoms at 6 months (one trial, n=330;	Attrition bias; long-term results differ from short-term trials	Treatment discontinuation due to adverse events: RR 1.48 (95% CI 0.50–4.35; 3 RCTs, n=1090), with	[51]

		certainty (one trial, n=330)			reported separately.		moderate certainty)		no clear difference versus placebo. Comprehensive reporting of all adverse-event types and serious events was unavailable.	
PEA / mixed chronic pain	Schweiger et al., 2024; systematic review/meta-analysis; 9 studies (742 patients)	Evidence synthesis of predominantly uncontrolled studies (8 of 9); not a comparative efficacy estimate	Micron-size oral PEA preparations	Varied across studies; no single dose can be assigned from the summary	At least 60 days; comparison of pain scores beyond the first month	Mostly uncontrolled; background therapies varied	Further reduction in reported pain intensity beyond 30 days	High clinical heterogeneity, concomitant treatments, uncontrolled designs; includes authors of the present review	No pooled comparative adverse-event estimate established from the review summary.	[33]
PEA / mixed CP	2023 systematic review / meta-analysis; 11 double-blind RCTs (774)	Systematic review / meta-analysis of double-blind RCTs; certainty or risk of bias: Not established from the reviewed source	PEA formulations varied; primarily oral	Range of daily doses in the included trials: Not established from the reviewed source	Not established from the reviewed source	Placebo or active comparator	Pooled reduction in pain intensity relative to controls	Mixed indications/formulations; no direct proof of target engagement	Adverse-event capture and reporting varied among the 11 RCTs; some trials reported no events, while others reported mild transient events. No pooled comparative safety estimate should be inferred.	[28]
PEA / mixed pain	2025 systematic review / meta-analysis; 18 RCTs (1,196)	Systematic review / meta-analysis; certainty or risk of bias: Not established from the reviewed source	Not established from the reviewed source	Regimens varied; no single pooled dose reported	Not established from the reviewed source	Control arms varied	Pain reduction signal	Heterogeneity and risk of bias Not established from the reviewed source	The review does not provide a sufficiently consistent pooled adverse-event estimate to establish comparative safety across formulations and indications.	[29]
um-PEA / fibromyalgia	retrospective study, 407 patients; add-on treatment	Observational; no certainty rating; authors include members of the present group	Ultramicronized PEA; oral	Not established from the reviewed source	Not established from the reviewed source	Add-on to existing therapy; control group: Not established from the	Not established from the reviewed source	Retrospective design; potential for bias; authored by members of the present group	Adverse events were reported by 36 treated patients (13.7% of the safety population), mainly gastrointestinal	[31]

						reviewed source			(diarrhoea, dyspepsia, bloating, constipation and vomiting); uncontrolled retrospective design.	
Curcuminoids / knee OA	2022 meta-analysis; 15 studies (1670)	Systematic review / meta-analysis; certainty or risk of bias: Not established from the reviewed source	Curcuminoid preparations; oral	Not established from the reviewed source	Short term; varied	Placebo / NSAIDs	Pain and WOMAC function outcomes improved in pooled comparisons	Study quality and heterogeneity; formulations not equivalent	Two of 15 studies reported no adverse events; the remaining 13 predominantly reported gastrointestinal symptoms. No significant difference versus placebo (RR 1.07, 95% CI 0.70–1.65); comparison with NSAIDs also not significant in the main analysis.	[21]
Turmeric extracts / knee OA	2021 systematic review; 16 RCTs (1810)	Systematic review of RCTs; moderate risk of bias (as reported)	Turmeric extracts; oral	Not established from the reviewed source	≤16 weeks	Placebo / NSAIDs	Pain and function improvement in pooled analysis	High heterogeneity; moderate risk of bias	Adverse-event rates were similar to placebo and approximately 12% lower than NSAID comparators in the pooled review; short trial duration limits long-term safety conclusions.	[20]
Nanocurcumin / DPN	[Primary RCT] randomized double-blind trial in type 2 diabetes (N: Not established from the reviewed source)	Primary RCT (single trial); certainty not graded	Nanocurcumin; oral	Not established from the reviewed source	16 weeks (not independently confirmed)	Placebo (not independently confirmed)	No improvement in pain or neuropathic outcomes versus placebo (not independently confirmed)	Single trial; results cannot be extended to other curcumin preparations	One participant in the nanocurcumin group discontinued because of itching (2.1%), versus none with placebo. No significant between-group	[63]

									differences in measured hepatic, renal or thyroid laboratory markers at 16 weeks.	
CoQ10 / painful DPN	2022 double-blind RCT (112)	Primary RCT (single double-blind trial); certainty not graded	CoQ10 oral add-on	100 mg every 8 h (300 mg/day)	8 weeks	Pregabalin 150 mg/day in both groups; placebo	Pain NRS improved versus placebo add-on	Single trial; does not establish monotherapy efficacy	The available study summary does not establish a numerical adverse-event comparison; absence of a statistically significant difference should not be interpreted as absence of events.	[55]
Omega-3 / mixed CP	2025 systematic review; 41 RCTs (3759)	Systematic review of RCTs; certainty: Not established from the reviewed source	EPA/DHA formulations varied	Not established from the reviewed source	Varied (including 1–6 months)	Placebo or active comparators	Pooled pain reduction (SMD −0.55; 95% CI −0.76 to −0.34); effect differs by condition [104].	High between-trial heterogeneity (I^2 = 87%); no statistically clear benefit in OA subgroup [104].	The pain-focused meta-analysis did not provide a pooled adverse-event estimate suitable for comparison across the 41 heterogeneous RCTs; safety cannot be inferred from the pooled pain outcome.	[45]
Magnesium / chronic pain	Systematic review (not a meta-analysis); studies: Not established from the reviewed source	Systematic review; certainty: Not established from the reviewed source	Magnesium; route varied (as stated in the manuscript text)	Not established from the reviewed source	Not established from the reviewed source	Not established from the reviewed source	Greatest benefit in migraine prophylaxis, less in some neuropathic pain conditions (as stated in the manuscript text)	Dosing regimens and routes vary across studies	Adverse events were inconsistently reported across the nine RCTs; the systematic review explicitly stated that no judgement on safety could be made.	[70]
Boswellia / knee OA	2024 systematic review; 9 RCTs (712)	Systematic review / meta-analysis; certainty: Not	Standardized Boswellia extracts	Not established from the	Not established from the	Control therapy	Pooled improvements in pain and function	Extract-specific; standardization and potential industry	The efficacy-focused systematic review does not	[79]

		established from the reviewed source		reviewed source	reviewed source			relationships warrant attention	establish a robust pooled comparative adverse-event estimate across heterogeneous extracts; no conclusion of equivalent safety is warranted.	
Ginger / OA	2015 meta-analysis; 5 RCTs (593)	Meta-analysis of RCTs; certainty: Not established from the reviewed source	Oral ginger preparations	Not established from the reviewed source	Not established from the reviewed source	Placebo	Small pooled pain/disability benefit	Withdrawal for adverse events more frequent than placebo	Withdrawals due to adverse events were more frequent with ginger than placebo (RR 2.33, 95% CI 1.04–5.22; five RCTs, n=593).	[82]
Ginger / knee OA	2020 systematic review; 7 trials (4 meta-analyzed)	Systematic review / meta-analysis; methodological quality described as poor	Oral or topical ginger	Not established from the reviewed source	Not established from the reviewed source	Placebo or standard care	Insufficient consistent evidence for knee pain/function	Methodological quality poor; differing delivery routes	The review did not establish a pooled comparative adverse-event estimate across oral and topical ginger preparations; safety reporting should not be interpreted as demonstrating absence of harm.	[83]
ALC / peripheral NP	2015 meta-analysis; 4 RCTs (523)	Meta-analysis of RCTs; certainty: (see Cochrane review [88])	Oral or sequential IM/oral ALC	Not established from the reviewed source	Not established from the reviewed source	Placebo	Pain VAS reduction in pooled analysis	Follow-up and certainty limitations; newer Cochrane findings require balance	Safety data were heterogeneous across the trials included in the meta-analysis; the later Cochrane review found low-certainty evidence and no clear overall difference in adverse-event withdrawals versus placebo.	[72]

ALC / DPN	2019 Cochrane review; trials and participants: Not established from the reviewed source	Systematic review (Cochrane); certainty for pain outcomes: very low	ALC; Not established from the reviewed source	Not established from the reviewed source	Not established from the reviewed source	Not established from the reviewed source	Effect on pain uncertain; certainty very low (as stated in the manuscript text)	Very low certainty; see also the meta-analysis [60]	In one placebo-controlled study, discontinuations due to adverse events occurred in 6/147 (4.1%) with ALC and 2/147 (1.4%) with placebo (P=0.17); reported events included headache, facial paraesthesia and gastrointestinal disorders. Other trials had incomplete reporting; overall certainty was low.	[73]
Vitamin D / FMS	2025 systematic review (7 studies), with four studies included in each pooled analysis	Systematic review/meta-analysis; substantial heterogeneity (pain I^2 = 89%)	Vitamin D supplementation; oral preparations in included trials	Daily and intermittent regimens varied among trials; no single pooled dose or administration frequency can be assigned. Wepner et al. used 1,200–2,400 IU/day; other schedules require consultation of the original trial reports.	8–49 weeks across the studies listed by Ilari et al.; individual treatment schedules differ.	Placebo or other controls, depending on trial	Pooled analyses suggest improvements in pain and fibromyalgia-related impact; findings are not definitive	Small pooled datasets; substantial heterogeneity; baseline vitamin D status and dose schedules differ	Safety outcomes were not pooled or consistently reported across the included studies; no conclusion on comparative adverse-event rates can be drawn from this review.	[93]
Vitamin D / chronic painful conditions	2015 Cochrane systematic review; 10 RCTs (811 participants)	Systematic review; heterogeneous clinical indications and interventions	Vitamin D supplementation; formulations/routes varied across trials	Daily, intermittent and bolus regimens varied among the included trials; no uniform daily dose can be	Treatment durations varied across the included trials; see original RCTs for study-specific schedules.	Placebo or control	No consistent analgesic benefit across chronic painful conditions	Differences in pain conditions, baseline vitamin D status, and dosing schedules; limited comparability	Adverse events and withdrawals were relatively infrequent, with no consistent difference between vitamin D and placebo across included	[92]

				inferred from the review.					trials (as reported by the review authors).	

Safety note: Potential interaction warnings and contraindications require individual product labeling and patient-level assessment. The absence of a serious adverse event in a short trial does not establish long-term safety.

Note. Formulations, doses and routes are not interchangeable: findings obtained with one preparation should not be extrapolated to others without supporting pharmacokinetic or clinical evidence. Studies authored by members of the present group are indicated in the study and evidence columns. When details are unavailable in the evidence synthesis, the table avoids inferring that they were absent from the primary studies.

Abbreviations: ALA, alpha-lipoic acid; ALC, acetyl-L-carnitine; CoQ10, coenzyme Q10; CP, chronic pain; DPN, diabetic peripheral neuropathy; FMS, fibromyalgia syndrome; N, number of participants; NP, neuropathic pain; NR, not reported; NRS, numeric rating scale; OA, osteoarthritis; PEA, palmitoylethanolamide; RCT, randomized controlled trial; um-PEA, ultramicronized palmitoylethanolamide; WOMAC, Western Ontario and McMaster Universities Osteoarthritis Index.
